# Case report: Congenital arterioportal fistula: An unusual cause of variceal bleeding in adults

**DOI:** 10.3389/fmed.2022.970254

**Published:** 2022-09-07

**Authors:** Junlin Xia, Jingwen Zhao, Bing Chang

**Affiliations:** ^1^Department of Gastroenterology, The First Affiliated Hospital, China Medical University, Shenyang, China; ^2^Department of Gastroenterology, Shengjing Hospital of China Medical University, Shenyang, China

**Keywords:** hepatic arterioportal fistula, gastrointestinal bleeding, portal hypertension, coil embolization, occlusion, ascites, angiography, arteriovenous fistula

## Abstract

The aberrant vascular connecting channel that forms between the portal vein and the hepatic artery is the essence of a hepatic arterioportal fistula. Congenital hepatic arterioportal fistula more frequently occurs in early childhood rather than in adults. We describe a rare instance of a large, isolated, congenital hepatic arterioportal fistula that was successfully treated following selective hepatic arteriography and transcatheter embolization. The patient presented with significant variceal bleeding when the fistula was discovered at the age of 73. The patient's condition improved during the brief postoperative follow-up period without a recurrence. Our research suggests that in older patients with portal hypertension and an unclear etiology, selective arteriography and embolization can provide a definitive diagnosis and successfully treat symptoms.

## Introduction

Hepatic arterioportal fistula (HAPF) is an uncommon kind of arteriovenous fistula. Congenital intrahepatic arterioportal fistulas (CIAPF) are typically numerous, diffuse, and diagnosed in childhood or infancy, with only a few occurrences detected in adults. In most cases, HAPF identified at maturity is assumed to have been acquired (1). Due to an increase in reports, hepatic arteriovenous fistulas in adults are becoming more widely recognized as a rare cause of portal hypertension. We encountered an older patient with severe variceal hemorrhage who was later diagnosed with CIAPF.

## Case report

A 73-year-old female patient with a history of unexplained intermittent hematochezia characterized by dark red stool 6 months before admission and no abnormalities on barium meal examination. Her hematochezia had deteriorated 3 days ago, with a volume of ~700 ml and hematemesis of ~200 ml, but with no abdominal pain or distension. Symptomatic treatments such as proton pump inhibitor, somatostatin, rehydration therapy, and blood transfusion therapy were given in the emergency room. The initial diagnosis was a massive upper gastrointestinal tract bleed. She had a 30-year history of hypertension, with her blood pressure remaining at 190/110 mmHg. She denied any history of liver illnesses such as hepatitis or cirrhosis, and any traumatic liver or abdominal intervention such as punctures or other procedures, which could be a secondary cause of HAPF. Social and family histories were unremarkable. Physical examination showed pale lid conjunctiva and nail bed, active bowel sounds, and abdominal tenderness. There was no facial darkness, no hepatomegaly or spider nevus, and no hepatosplenomegaly.

**Figure 1:**
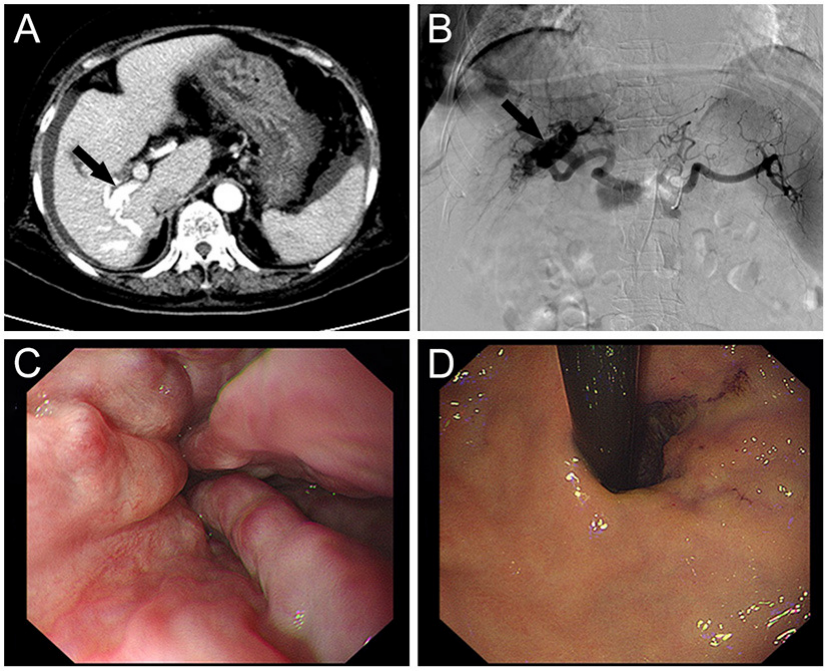
**(A)** Enhanced CT shows early portal vein visualization in the arterial phase suggesting hepatic arterioportal fistula (black arrow). **(B)** Selective hepatic artery angiography demonstrated the dilated right hepatic arteries (black arrow). The left hepatic artery was normal. **(C)** Endoscopy display the tortuous varices with dilatation and positive RC sign in esophagus. **(D)** Endoscopy display the tortuous varices with dilatation and positive RC sign in gastric fundus.

Laboratory results were as follows (Table 1). The levels of alanine aminotransferase (ALT), alkaline phosphatase (ALP), gamma-glutamyltransferase (GGT), serum total bilirubin, and serum direct bilirubin were within normal limits. All hepatitis virus markers and autoantibodies (antinuclear, anti-smooth muscle, anti-neutrophil cytoplasmic, anti-soluble liver antigen/anti-hepatopancreatic, anti-actin, liver kidney microsomal 1 antibody, liver cytosol antigen type 1 antibodies) were also negative. Serum alpha-fetoprotein levels were normal. Computed tomography (CT) scan revealed a morphologically sound liver with a smooth surface. An early enhancement, contrast-enhanced CT showing simultaneous imaging of the artery and the distal right trunk of the portal vein (Figure 1A), indicated a probable arterioportal fistula. The intrahepatic and extrahepatic bile ducts did not dilate. There were esophageal and gastric fundus varices. The spleen was uniformly dense and modest in size. The left kidney had shrunken to the point that just a small amount of residual tissue remained, and a small hypodense nodular shadow was detected in the location of the left adrenal gland. There was a significant quantity of ascites present.

**Table 1:** Laboratory findings before surgical.

**Test**	**Result**	**Reference range**
Red blood cell (× 10^12^ /*L*)	2.68	3.80–5.10
Leukocytes (× 10^9^ /*L*)	5.46	5–34
Platelets (× 10^9^ /*L*)	118	150–400
Hemoglobin (g/L)	65	100–140
Mean corpuscular volume (fL)	84.0	82.0–100.0
MCH (pg)	24.3	27.0–34.0
MCHC (g/L)	289.0	316.0–354.0
Albumin (g/L)	34.6	35.0–50.0
Urea (mmol/L)	8.0	2.5–6.1
Prothrombin time (s)	17.8	11.0–14.3
Prothrombin activity (%)	56	80–120%
INR	1.50	0.82–1.15

MCH, mean corpuscular hemoglobin; MCHC, mean corpuscular hemoglobin concentration; INR, International normalized ratio.

After integrating the patient's laboratory results and medical history and ruling out any secondary causes of HAPF, the diagnosis of CIAPF was confirmed with the help of CT and selective hepatic arteriography. The differentiation of CIAPF from Budd-Chiari syndrome and portal-hepatic venous fistula deserves to be considered. Signs such as aneurysm-like dilatation of the hepatic vein and accessory hepatic veins, portal hypertension, stenosis of the inferior vena cava and hepatic veins are helpful in the diagnosis of Budd-Chiari syndrome, while portal-hepatic fistula is abnormal traffic between the portal and hepatic veins that can be recognized by CT and arteriography.

Following successful Seldinger technique puncture of the right femoral artery, a 5-F arterial sheath and right hepatic artery catheter were delivered for selective hepatic arteriography, which revealed a significant arterioportal fistula in the right lobe of the liver (Figure 1B). Two 3 and two 4 mm micro steel coils were placed along the microcatheter following the introduction of the microcatheter to clarify the blood-supplying artery. After repeated angiography, a small fistula remained, but the portal vein fistula vanished after reapplication of 1/4 of a 500–700 μm gelatin sponge. The tube was removed, and hemostasis was achieved by compressing the wound for 10 min before applying pressure dressings.

**Figure 2:**
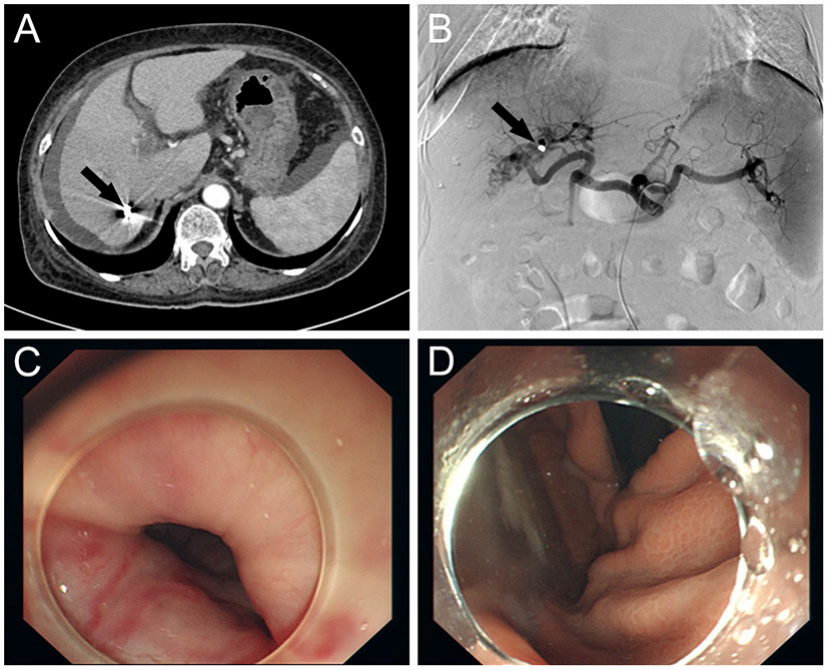
**(A)** CT scan of the abdomen: CT shows occlusion of the hepatic arterioportal fistula after embolization (black arrow) and reduction of ascites. **(B)** Selective hepatic artery angiography showing complete closure of the fistula (black arrow) between the right hepatic artery and the right portal vein branch after coil embolization. **(C)** Improvement of varicose veins after endoscopic sclerotherapy. **(D)** Improvement of varicose veins after endoscopic sclerotherapy.

The patient's vital signs remained steady during the procedure, and there was no considerable discomfort. Endoscopic sclerotherapy was performed, and four tortuous varices with dilatation and positive red color (RC) signs were seen throughout the esophagus (Figures 1C,D). After treatment, the varices improved. The patient recovered well after embolization and sclerotherapy (Figure 2), and no further upper gastrointestinal bleeding and any other complications associated with portal hypertension occurred throughout the 6-months follow-up period (Supplementary Figure S1).

## Discussion

Fistulas come in a variety of types, depending on the etiology, vessels involved, size, and location. Acquired arterioportal fistulas are most commonly caused by abdominal trauma or iatrogenic injury, whereas congenital intrahepatic arterioportal fistula has no hereditary susceptibility according to reported cases (1). The youngest case of CIAPF was found at 20 weeks of gestational age and diagnosed by Doppler ultrasonography at birth (2), while the oldest case was diagnosed at 74 years of age (3). According to the afferent supplying vessels, Norton et al. divided CIAPF into three types. Type 1, a unilateral IAPF, is supplied by only one of the right, left, or main hepatic artery. Type 2, bilateral lesions, indicate that the afferent supplying vessels are from both of the parent hepatic arteries or their branches. Complex lesions are type 3 consist typically of a plexiform vascular nidus with multiple feeding arteries, including supply from arteries other than the hepatic arteries (e.g., gastric artery) (4). CIAPF is an uncommon cause of portal hypertension in babies that takes several months to develop and might be asymptomatic at the time of diagnosis (2, 5), with symptoms presenting in approximately 70% of cases before the age of 2 years (4). Adult patients may present with more severe stomach pain, variceal bleeding, hepatic encephalopathy (3), spontaneous hepatic hemorrhage (6), and other symptoms comparable to those seen in infants, such as upper gastrointestinal bleeding, ascites, and hepatosplenomegaly (7). There is some heterogeneity in the onset of clinical symptoms in different types of CIAPF classified by Norton et al. (4), which could be related to the size of the fistula and the intricacy of its blood supplying arteries (Table 2). Most patients (*n* = 50) are diagnosed in infancy, and fistulas discovered in adulthood are typically difficult to diagnose, with the diagnostic basis consisting of a thorough history and radiological examination. Only 11 adult patients have been found, with 90.9% (*n* = 10) of the adult cases belonging to type 1, while the remaining adult case did not specify the artery, and it was unclear to which type it belonged.

**Table 2:** Review of reported cases based on “Norton-Jacobson” classification.

**Type**	**Percentage (number)**	**Median age of diagnosis (range)**	**Treatment**
1^*^	49.2% (*n* = 30)	20 mo (range: 1 day−74 years)	Embolization alone 66.7% (*n* = 20)
			Surgical alone 23.3% (*n* = 7)
			Surgical + embolization 10.0% (*n* = 3)
2^*^	21.3% (*n* = 13)	5 mo (range: 5–14 years)	Embolization alone 61.5% (*n* = 8)
			Surgical alone 7.7% (*n* = 1)
			Surgical + embolization 30.8% (*n* = 4)
3^*^	16.4% (*n* = 10)	5 mo (range: 14 days−3 years)	Embolization alone 70.0% (*n* = 7)
			Surgical alone 0.0% (*n* = 0)
			Surgical + embolization 30.0% (*n* = 3)
Not mention	13.1% (*n* = 8)	None	None

1^*^: supplied by only one of the right, left or main hepatic artery.

2^*^: include supply from both of the parent hepatic arteries or their branches.

3^*^: consist typically of a plexiform vascular nidus with multiple feeding arteries, including supply from arteries other than the hepatic arteries (e.g., gastric artery).

Not mention: These cases, as well as some others not included in this classification, did not expressly describe the blood supply artery, so their type cannot be determined.

The vascular architecture framework is commonly identified by CT, and HAPF is determined by early enhancement of the lesion in the arterial phase (4). Angiography is the gold standard for diagnosing HAPF, and it may detect multiple forms of the disease and function as a diagnostic and therapeutic tool (8).

Interventional radiology is rapidly becoming more critical as modern medicine advances. Transcatheter arterial embolization has become the most prevalent treatment technique because of lower cost, less invasiveness, less pain, improved targeting of the supplying artery, and excellent results (5). In HAPF patients, embolization has become the first-line treatment (Table 2).

Surgical interventions such as fistulotomy and direct vascular repair, arterial ligation, hepatectomy, liver transplantation, or a combination of surgical and interventional treatment are required in complex cases with multiple failed transcatheter arterial embolization, recurrent, refractory, or multiple fistulas (9). With today's therapeutic options, patients have a greater chance of survival, with only 3.3% (*n* = 2) dying from this disease. A total of 96.7% (*n* = 59) of patients were effectively treated. The prognosis is improved in the short term.

## Conclusion

The patient was an elderly patient who appeared with more severe gastrointestinal bleeding despite the fact that congenital hepatic arteriovenous fistulas are uncommon and often present in infancy. This was the case even though there was no sign of abnormal liver function or other liver diseases. Medical history and associated tests resulted in a precise diagnosis and efficient treatment. The study adds to the knowledge of the clinical presentation and treatment of this uncommon condition. In prior reported instances, serious gastrointestinal bleeding has presented less frequently. We must continue to monitor the patient's long-term prognosis since, despite the favorable short-term follow-up outcomes, the patient's older age caused the function of several systems to diminish with age. However, it is challenging for us to track the patient for an extended length of time due to geographic restrictions and changes in patient contact information. Still, we won't hold back in our pursuit of predictive data pertaining to her.

And our patient reported the following about her experience: “I was made aware of the initial diagnosis that underwent further testing might be validated and treated as the reason of my clinical presentation as well as my concerns regarding the disease's management during my clinical therapy. I routinely underwent post-operative assessment and treatment since I was aware of the possibility of postoperative recurrence. After a stable 6-months evaluation, no gastrointestinal bleeding, etc., occurred, and I was pleased with the outcomes.”

## Data availability statement

The original contributions presented in the study are included in the article/Supplementary material, further inquiries can be directed to the corresponding author.

## Ethics statement

Ethical review and approval was not required for the study on human participants in accordance with the local legislation and institutional requirements. Written informed consent from the [patients/participants OR patients/participants legal guardian/next of kin] was not required to participate in this study in accordance with the national legislation and the institutional requirements. The patients/participants provided their written informed consent to participate in this study.

## Author contributions

JX drafted the manuscript, performed the selection and organization of literature, and prepared the figures and the table. JZ and BC helped to revised the manuscript. BC carried out the design of this review and revised the manuscript. All authors contributed to this manuscript and approved the final manuscript.

## Funding

This work was also supported by a grant from Shenyang science and technology plan fund project (20-205-4-094).

## Conflict of interest

The authors declare that the research was conducted in the absence of any commercial or financial relationships that could be construed as a potential conflict of interest.

## Publisher's note

All claims expressed in this article are solely those of the authors and do not necessarily represent those of their affiliated organizations, or those of the publisher, the editors and the reviewers. Any product that may be evaluated in this article, or claim that may be made by its manufacturer, is not guaranteed or endorsed by the publisher.

## Abbreviations

HAPF: hepatic arterioportal fistula
CIAPF: congenital intrahepatic arterioportal fistulas
ALT: alanine aminotransferase
ALP: alkaline phosphatase
GGT: gamma-glutamyltransferase
CT: computed tomography
RC sign: red color sign.
