# Supplementary figures and images for: Case report: Congenital arterioportal fistula: An unusual cause of variceal bleeding in adults
Source: Front Med (Lausanne). 2022 Sep 7;9:970254. doi: 10.3389/fmed.2022.970254 (PMC9489925; doi:10.3389/fmed.2022.970254)

**Fig. S1**

Case Progress Timeline

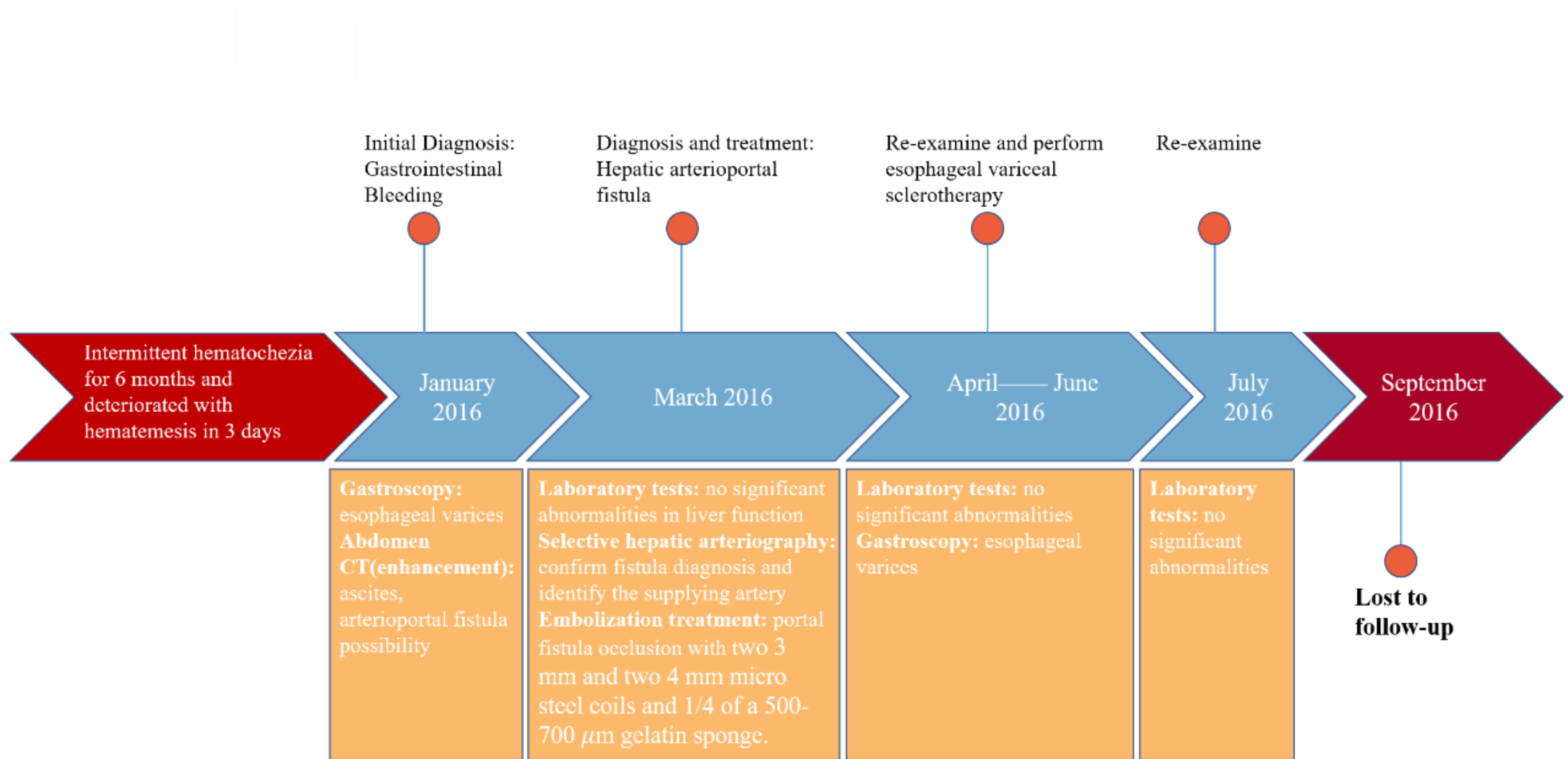

Supplement: Supplementary file 1 [file Image_1.pdf]
